# Dyspnea after a first episode of pulmonary embolism: prevalence, predictors and long-term associations with health-related quality of life

**DOI:** 10.3389/fcvm.2025.1595705

**Published:** 2025-07-07

**Authors:** Inge Kirchberger, Simone Fischer, Thomas M. Berghaus, Jakob Linseisen, Christine Meisinger

**Affiliations:** ^1^ Epidemiology, Faculty of Medicine, University of Augsburg, Augsburg, Germany; ^2^ Department of Cardiology, Respiratory Medicine and Intensive Care, University Hospital Augsburg, Augsburg, Germany; ^3^ Medical Faculty, Ludwig-Maximilians-University München, Munich, Germany; ^4^ Institute for Medical Information Processing, Biometry and Epidemiology—IBE, LMU Munich, Munich, Germany

**Keywords:** pulmonary embolism, dyspnea, quality of life, depression, anxiety

## Abstract

**Introduction:**

The predictors and consequences of dyspnea after pulmonary embolism (PE) are only rarely investigated. The present study aimed to characterize dyspnea and its associated factors in patients with incident PE up to 2 years after hospital discharge.

**Methods:**

Data from the German “Lungenembolie Augsburg (LEA)” cohort study were used. Baseline characteristics of adult patients with a first episode of acute PE were collected during hospital stay. Participants completed postal questionnaires 3, 6, 12, and 24 months after their PE. Dyspnea was assessed using the Chronic Respiratory Questionnaire (CRQ) and the Pulmonary Embolism Quality of Life Questionnaire (PEmb-QoL) was used to measure health-related quality of life (HRQOL). Linear mixed models were used to determine the variables associated with dyspnea.

**Results:**

Out of 503 patients (55% male, mean age 62.8 ± 14.6 years), 45%–64% of the participants had dyspnea at any time point. No significant change of dyspnea over time was found. Body mass index (estimate −0.04, 95% CI −0.06 to −0.02, *p* = 0.0002), symptoms of depression (estimate −0.11, 95% CI −0.15 to −0.07, *p* < 0.0001), symptoms of anxiety (estimate −0.08, 95% CI −0.11 to −0.04, *p* < 0.001), and FEV_1_ values (estimate 0.35, 95% CI 0.10–0.61, *p* = 0.0060) were significantly associated with the CRQ dyspnea score. Furthermore, dyspnea had significant and strong adverse associations with all subscales of the PEmb-QOL (*p* < 0.0001).

**Discussion:**

Dyspnea is a common and persisting complaint after PE. Symptoms of depression and anxiety are strongly related with dyspnea and dyspnea is significantly associated with impaired HRQOL.

## 1 Introduction

Acute pulmonary embolism (PE) is a serious clinical presentation of venous thromboembolism (VTE), and the third most common cardiovascular condition after myocardial infarction and stroke (1). While mortality rates in the acute phase are decreasing, incidence rates of PE are rising in the ageing societies (2–8). The long-term course of PE is characterized by a considerable proportion of patients who experience persisting dyspnea, limited exercise capacity, impaired functional status, or poor health-related quality of life (HRQOL) (9–15). This heterogenous condition is also termed “post-pulmonary embolism syndrome” (16, 17). In addition, mental problems such as depression or anxiety are common among survivors of acute PE (18, 19).

Dyspnea has been identified as a common long-term sequalae of PE affecting up to 50% of the patients (11, 14, 15, 20, 21, 22). It was shown to be significantly associated with impaired generic and disease-specific HRQOL in a few cross-sectional studies with samples of approximately 200 patients or less (11, 14, 23), whereas long-term investigations of this association are rare and are only conducted in smaller patient samples (21). Furthermore, it is still unclear what the risk factors of developing persisting dyspnea after acute PE are. Available studies indicated that female sex, advanced age, smoking history, cardiopulmonary comorbidity, higher BMI, depression, recurrent VTE, diffusion capacity of the lung for carbon monoxide, persistent perfusion defects, predicted VO2 peak <80%, pulmonary artery systolic pressure, chest pain, breathing variability and follow-up-time may play a role in the occurrence of persisting dyspnea (20, 21, 24–26), but the identified variables vary considerably across the studies. Other investigators were even not able to find any significant independent predictors of dyspnea (14).

Hence, the objective of the present study was to characterize dyspnea in patients with PE up to 2 years after hospital discharge, to identify factors associated with dyspnea and to determine the association between dyspnea and HRQOL.

## 2 Methods

### 2.1 Design and study population

The study sample consisted of participants of the “Lungenembolie Augsburg” (LEA) study. The LEA study is a long-term observational cohort study including adult patients with incident or recurrent confirmed PE diagnosis based on multidetector CT pulmonary angiography or ventilation–perfusion lung scanning who were hospitalized and treated at the University Hospital Augsburg, Germany. The study design has been described in detail by Meisinger et al. (27).

During their hospital stay, a baseline personal interview was conducted by study nurses. It covers information on socio-demographics, risk factors, and comorbidities. In addition, the patients completed a self-administered questionnaire on mental health. After discharge, study participants were requested to complete postal questionnaires after 3, 6, and 12 months in the first year, thereafter in yearly intervals up to 60 months.

**Figure 1:**
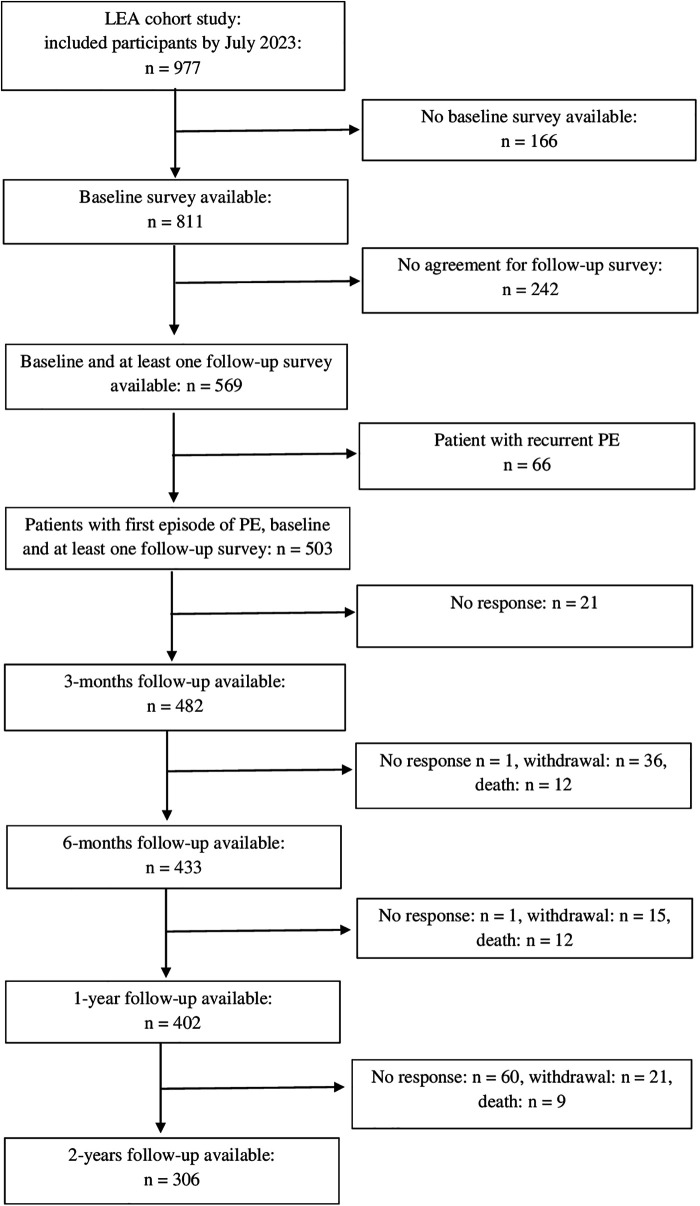
Flow chart of the study.

In total, 977 patients were included in the study between July 2017 and July 2023. The present analyses are based on data from 503 study participants with a first episode of PE who were followed up to two years post PE. Of these, 262 participants completed all four follow-up surveys. Figure 1 shows the flow chart of the study.

### 2.2 Clinical and socio-demographic characteristics

Clinical characteristics of the study participants were extracted from the medical records. Information on uni- versus bilateral PE presentation was collected. If a CT pulmonary angiography was available, information on the presence of central thrombi and infiltrates was noted. For patients who had a pulmonary function test during their hospital stay, the forced expiratory volume in 1 s (FEV1) was included in the data analysis. The Simplified Pulmonary Embolism Severity Index (sPESI) was calculated based on the patient's age, history of cancer, chronic cardiopulmonary disease, heart rate, systolic blood pressure and arterial oxygen saturation to classify patients into high (≥1) or low (0) PE-related risk of death (28). Furthermore, information on prior and current concomitant diseases, PE treatment and duration of hospitalization was extracted from the medical records.

Information on age, gender, marital status, school and professional education, risk factors such as smoking and body mass index, as well as prior and current diseases (e.g., asthma, COPD, cancer, depression) were obtained from the personal interview. School and professional education were classified according to the International Standard Classification of Education (ISCED) system into the levels 1 (lowest level) to 5 (highest level) (29). For the multivariable analyses ISCED categories 1 and 2 as well as the categories 4 and 5 were collapsed. For the analysis, self-reported information on comorbidities was only considered if the information was not available from the medical records.

### 2.3 Survey data

The postal follow-up assessments at 3 months, 6 months, 1 year and 2 years after the acute PE event included standardized questionnaires to assess dyspnea, HRQOL, and mental health.

Dyspnea was assessed in three different ways. First, the 5-item dyspnea subscale of the self-administered Chronic Respiratory Questionnaire (CRQ) (30) was used. These items request the intensity of dyspnea during the past 3 days on a scale with seven verbal response options (no dyspnea to extremely strong dyspnea) in five different situations, namely while walking, socializing, doing household chores, personal hygiene activities such as bathing, dressing or eating, and when having emotions (e.g., anger). The summary score of these items ranges between 1 and 6 with higher scores indicating less impairment.

Furthermore, two items from the PEmb-QoL questionnaire ask about the frequency of breathlessness during the past 4 weeks (never, less than once a week, about once a week, several times a week, every day) and its intensity (none, very slight, slight, quite a bit, serious, very serious). Higher scores indicate a higher frequency (range 1–5) or stronger intensity (range 1–6).

The PEmb-QoL questionnaire was also used to assess PE-specific HRQOL (31). It includes 38 items in six subscales (Frequency of complaints, intensity of complaints, activities of daily living limitations, work-related problems, social limitations, emotional complaints). Subscale scores were transformed into a scale ranging from 0–100 with higher scores indicating worse HRQOL (32, 33). Since questions about dyspnea were included in the PEmb-QoL subscales “Frequency of complaints” and “Intensity of complaints” these two subscales were not used as outcome measures.

Symptoms of depression and anxiety were assessed using the Hospital Anxiety and Depression Scale (HADS-D), a self-administered and validated German version of the HADS (34–36). The HADS-D consists of two subscales with seven items each which measure depression and anxiety. The subscale scores range between 0 and 21 with higher scores indicating more severe symptoms of depression or anxiety. The subscale scores can also be classified into four categories: 0–7 (no depression or anxiety), 8–10 (mild depression or anxiety), 11–14 (moderate depression or anxiety) and 15–21 (severe depression or anxiety).

### 2.4 Data analysis

Multivariable linear mixed models with random intercepts were used to investigate the association between the CRQ dyspnea score (dependent variable) and clinical, sociodemographic, health-related variables, and follow-up time point (independent variables). Interaction terms between follow-up time point and all other independent variables were tested in the full model but were not significant and therefore not included in the final model.

Similarly, the association between the PEmb-QOL subscales “Activities of daily living limitations”, “Work-related problems”, “Social limitations”, “Emotional complaints” (dependent variables), and the CRQ dyspnea score (independent variable), adjusted for confounding variables (e.g., clinical, sociodemographic, health-related variables, follow-up time point) was determined using multivariable linear mixed models with random intercepts. Interaction terms between CRQ dyspnea score and follow-up time were included in the full models but were not significant and therefore not considered in the final models.

As sensitivity analyses, the above-mentioned analyses were repeated using the PEmb-QOL items on dyspnea frequency and intensity instead of the CRQ dyspnea score.

In all multivariable analyses, potentially confounding variables were selected based on directed acyclic graphs (37). For statistical tests an alpha level of 0.05 was defined. Statistical analyses were performed using SAS Version 9.4.

## 3 Results

### 3.1 Sample characteristics

The demographic and clinical characteristics of the participants are shown in Table 1. The median age of the 503 enrolled participants at the time of the PE event was 64 years (25% quantile: 55 years, 75% quantile: 74 years) with 55% men and 45% women. The median duration of hospitalization was 9 days (25% quantile: 6 days, 75% quantile: 13 days), and the median body mass index was 28.3 kg/m^2^ (25% quantile: 24.7 kg/m^2^, 75% quantile: 33.0 kg/m^2^). The median FEV1 was 2.3 L (25% quantile: 1.8 L, 75% quantile: 3.1 L) in 188 participants who had a pulmonary function test.

**Table 1:** Sample characteristics at baseline (*n* = 503).

Variables	N total	Total sample (*n* = 503)	%
		N	
Gender	503		
Male		275	54.7
Female		228	45.3
Education	491		
ISCED 1		4	0.8
ISCED 2		34	6.9
ISCED 3		313	63.8
ISCED 4		39	7.9
ISCED 5		101	20.6
Marital status	503		
Married		321	63.8
Single		70	13.9
Divorced		46	9.2
Widowed		66	13.1
Living alone	502	167	33.3
Respiratory diseases (ever diagnosed)			
Asthma	503	57	11.3
COPD	503	36	7.2
Pulmonary emphysema	503	11	2.2
Pneumonia	503	105	20.9
Sleep apnea	503	66	13.1
Interstitial lung disease	503	7	1.4
Other conditions (ever diagnosed)			
Hypertension	503	271	53.9
Heart failure	503	45	9.0
Diabetes	503	60	11.9
Inflammatory bowel disease	503	25	5.0
Neuromuscular disease	503	25	5.0
Autoimmune disease	503	46	9.2
Chronic hepatitis	503	5	1.0
Chronic kidney disease	503	37	7.4
Depression	503	63	12.5
Thrombophilia	503	16	3.2
Varicosis	502	129	25.7
Cancer treatment in the past 12 months	503	76	15.1
Smoking status	499		
Smoker		44	8.8
Ex-Smoker		202	40.5
Never smoker		253	50.7
sPESI	468		
Low risk (score = 0)		207	44.2
High risk (score ≥ 1)		161	55.8
PE location	491		
Unilateral		115	23.4
Bilateral		376	76.6
Pulmonary CTA	437		
Central thrombi		196	44.9
No central thrombi		241	55.1
Pulmonary CTA	426		
Infiltrates		186	43.7
No infiltrates		240	56.3
Treatment			
Intensive Care Unit	503	197	39.2
Ventilation	501	36	7.2
Anticoagulation	500	492	97.8
Embolectomy	502	3	0.6
Antibiotics	501	239	47.5
Thrombolysis	503	33	6.6
Depression^a^	503		
No		413	82.1
Mild		45	9.0
Moderate		29	5.8
Severe		16	3.2
Anxiety^a^	503		
No		393	78.1
Mild		69	13.7
Moderate		35	7.0
Severe		6	1.2

ISCED, international standard classification of education; COPD, chronic obstructive pulmonary disease; sPESI, simplified pulmonary embolism severity index; PE, pulmonary embolism; CTA, computed tomography angiography.

^a^ Hospital Anxiety and Depression Scale.

Compared with participants who were excluded for missing follow-up, the analyzed sample consisted of significantly more men, had less often past diagnoses of cancer and depression, had lower PESI scores and a shorter hospitalization.

### 3.2 Dyspnea within 2 years after hospital discharge

The five single items of the CRQ dyspnea scale showed that dyspnea was most common when walking or doing household chores (see Supplementary Figure S1). The percentage of individuals who had any intensity of dyspnea in the past 3 days during the follow-up time course ranged between 44.9% (during social activities 6 months after acute PE) and 63.6% (during household chores 3 months after acute PE). The percentage of persons experiencing at least moderate dyspnea intensity ranged between 13.1% (during social activities 2 years after acute PE) and 32.1 (during household chores 6 months after acute PE).

The PEmb-QOL items on frequency and severity of dyspnea during the past four weeks showed that between 55.0% and 57.2% of the participants had dyspnea at least once a week during the 2 year-follow-up, with 5.5% to 13.9% having dyspnea every day (see Supplementary Figure S2). The percentage of participants who had at least very slight dyspnea ranged between 57.1% (2 years after acute PE) and 62.8% (6 months after acute PE). The proportion of individuals who experienced “quite a bit” to “very serious” dyspnea ranged between 16.6% (2 years after acute PE) and 22.2% (6 months after acute PE).

**Figure 2:**
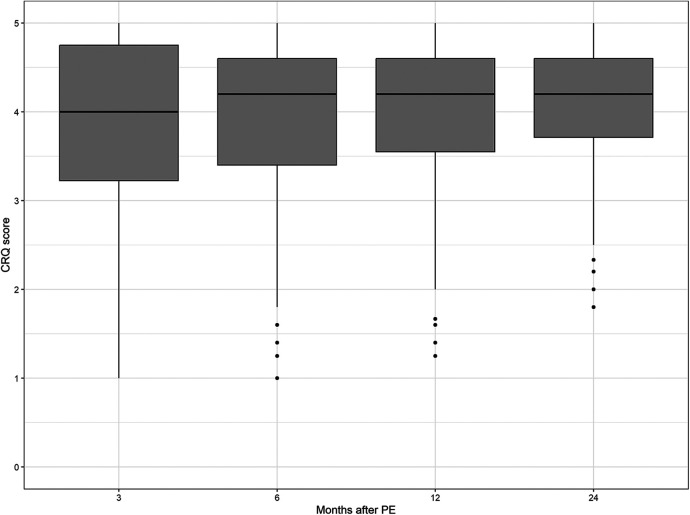
Chronic respiratory questionnaire scores over time. No significant differences between follow-up time points (*p* = 0.4986, Kruskal–Wallis test).

**Figure 3:**
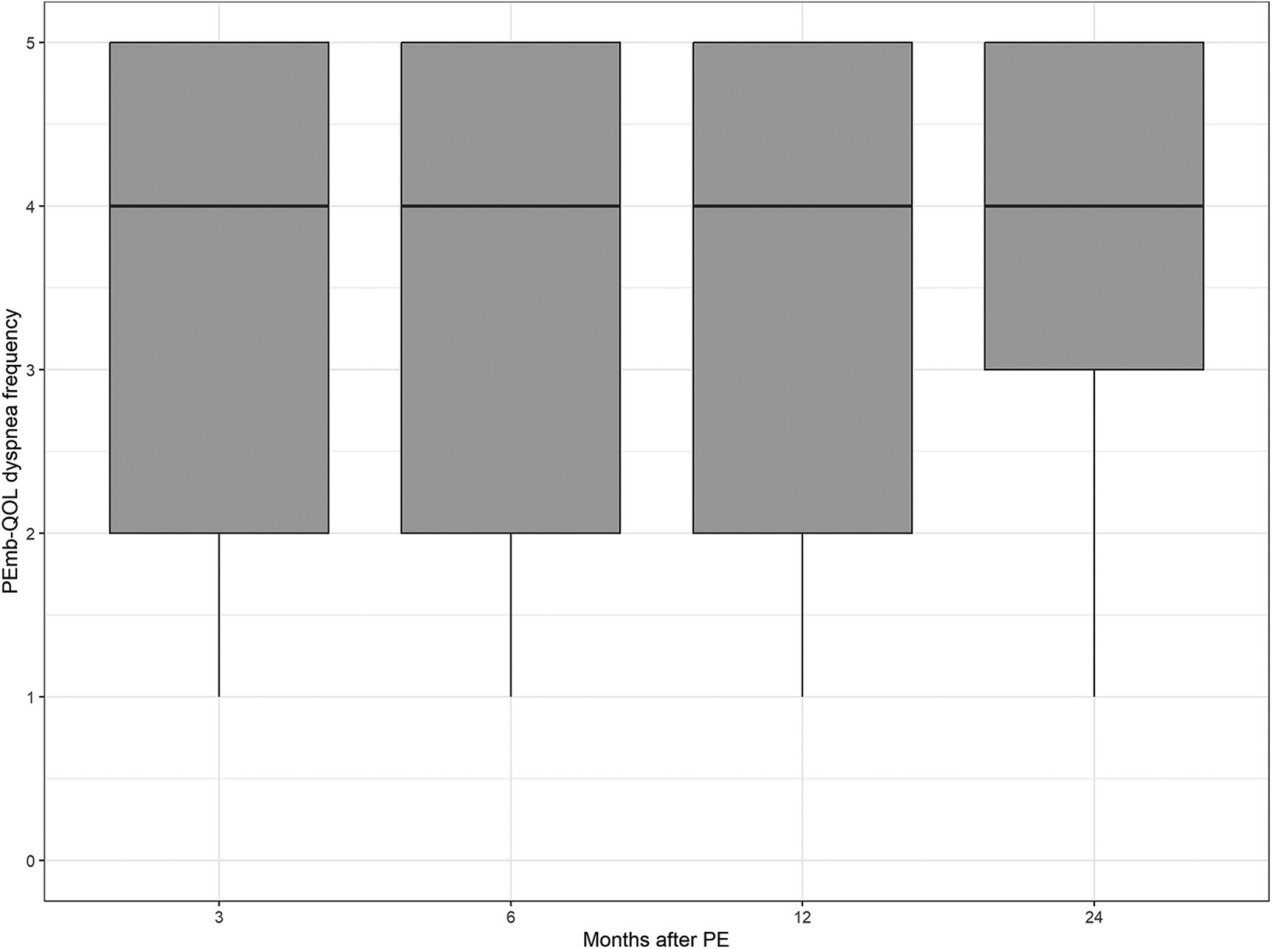
Dyspnea frequency according to the pulmonary embolism quality of life questionnaire over time. No significant differences between follow-up time points (*p* = 0.5767, Kruskal–Wallis test).

**Figure 4:**
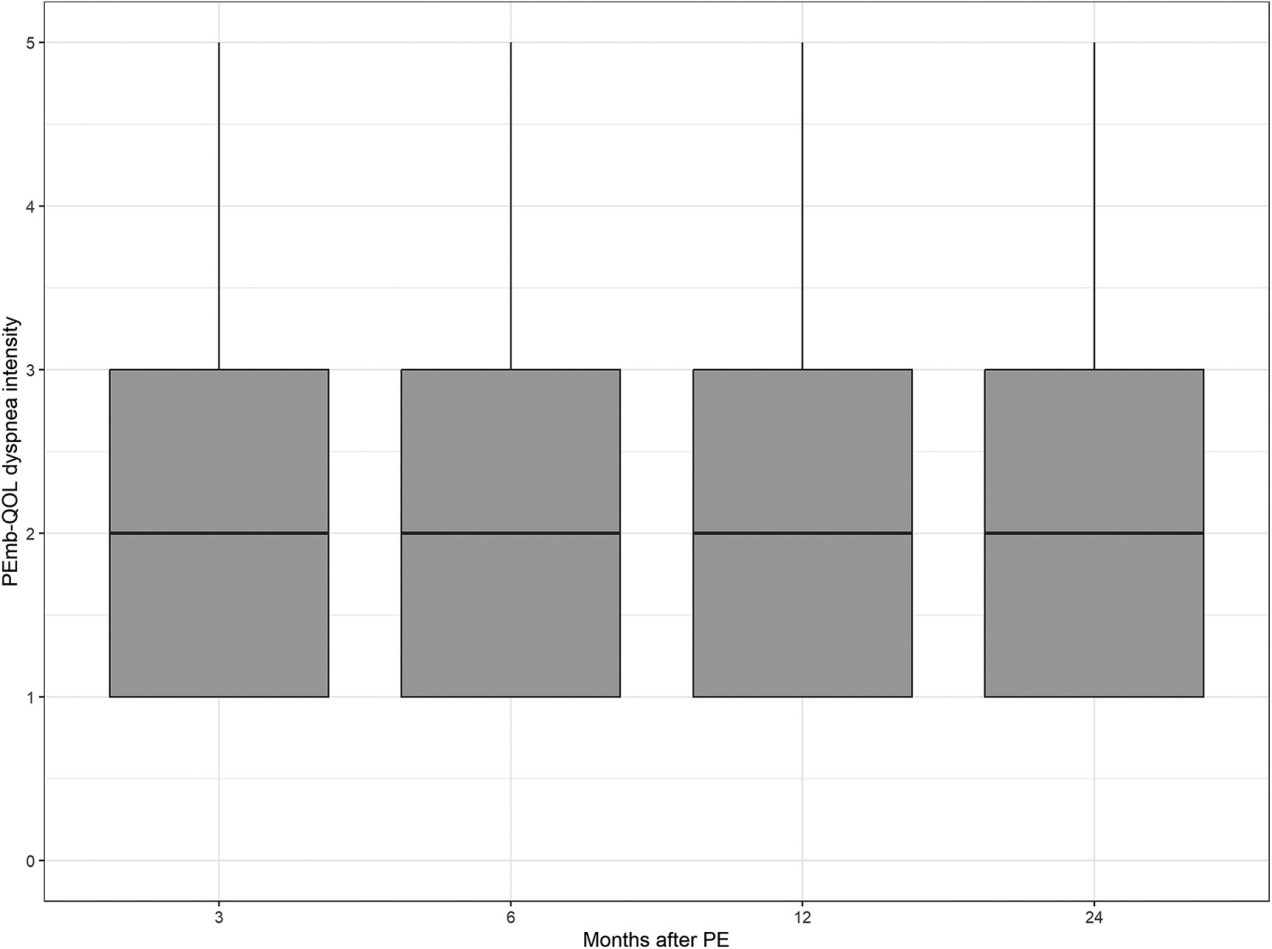
Dyspnea intensity according to the pulmonary embolism quality of life questionnaire over time. No significant differences between follow-up time points (*p* = 0.2732, Kruskal–Wallis test).

The median values of the CRQ dyspnea score and the PEmb-QOL items on frequency and severity of dyspnea did not differ significantly between the patient samples at each follow-up point (see Figures 2–4 and Supplementary Table S1).

### 3.3 Factors associated with dyspnea

Since clinical characteristics may be relevant factors associated with dyspnea, the analysis included results of pulmonary function tests and CT pulmonary angiography which were not performed in all participants, leading to a reduced analysis sample of 156 patients. Compared with the total sample, this subgroup was characterized by a lower proportion of patients with a history of thrombophilia or cancer, and a higher proportion of patients with a history of heart failure or sleep apnea. Moreover, this subgroup had a significantly higher BMI and a longer duration of hospitalization.

The results of the mixed models showed that higher CRQ dyspnea scores (indicating less impairment by dyspnea) were significantly associated with low body mass index (estimate −0.04, 95% CI −0.06 to −0.02, *p* < 0.001), less symptoms of depression (estimate −0.11, 95% CI −0.15 to −0.07, *p* < 0.0001), less symptoms of anxiety (estimate −0.08, 95% CI −0.11 to −0.04, *p* < 0.001) and high FEV1 (estimate 0.35, 95% CI 0.10–0.61, *p* < 0.01) (see Table 2). The follow-up time points were not significantly related with the CRQ dyspnea scores, therefore a stability of dyspnea within the interval of 3 months to 2 years after the acute PE can be assumed.

**Table 2:** Variables associated with dyspnea (chronic respiratory questionnaire): results of the mixed models (*n* = 156, 442 observations).

Variables	Dyspnea^a^	*p*-value
	Estimate (lower–upper CI)	
Female gender^b^	−0.007 (−0.40–0.39)	0.9729
Age	0.01 (−0.001–0.03)	0.0693
Education^c^		
ISCED 3	0.08 (−0.54–0.71)	0.7929
ISCED 4,5	0.58 (−0.11–1.27)	0.0973
Follow-up^d^		
6 months	−0.12 (−0.29–0.04)	0.1329
12 months	−0.05 (−0.19–0.14)	0.7937
24 months	0.01 (−0.17–0.19)	0.9088
History of cancer	0.03 (−0.39–0.46)	0.8721
History of asthma	−0.49 (−1.18–0.20)	0.1606
History of COPD	−0.41 (−0.95–0.13)	0.1344
Smoking^e^		
Current smoker	−0.10 (−0.53–0.55)	0.9612
Ex-smoker	−0.15 (−0.44–0.15)	0.3341
Body Mass Index [kg/m^2^]	−0.04 (−0.06–−0.02)	**0** **.** **0002**
Symptoms of depression^f^	−0.11 (−0.15–−0.07)	**<**.**0001**
Symptoms of anxiety^f^	−0.08 (−0.11–−0.04)	**<**.**0001**
Duration hospitalization	−0.02 (−0.04–0.001)	0.0691
sPESI score ≥1^g^	0.19 (−0.13–0.52)	0.2457
Bilateral PE localization	0.38 (−0.13–0.89)	0.1461
Central thrombi	−0.05 (−0.39–0.29)	0.7814
Infiltrates	−0.03 (−0.36–0.30)	0.8413
FEV_1_ [l]	0.35 (0.10–0.61)	**0**.**0060**

Bold type indicates significant *p*-values.

CI, confidence interval; ISCED, international standard classification of education; COPD, chronic obstructive pulmonary disease; PE, pulmonary embolism; sPESI, simplified pulmonary embolism severity index.

^a^ Chronic Respiratory Questionnaire, continuous score.

^b^ Reference: men.

^c^ Reference: ISCED 1,2.

^d^ Reference: 3-months follow-up.

^e^ Reference: never smoker.

^f^ Hospital Anxiety and Depression Scale continuous subscale scores.

^g^ Reference: sPESI score = 0.

**Table 3:** Association between dyspnea (chronic respiratory questionnaire) and the PEmbQol subscales: results of the mixed models (*n* = 440).

Variables	Activities of daily living limitations		Work-related problems		Social limitations		Emotional complaints	
	Estimate (lower–upper CI)	*p*-value	Estimate (lower–upper CI)	*p*-value	Estimate (lower–upper CI)	*p*-value	Estimate (lower–upper CI)	*p*-value
Dyspnea^a^	11.41 (−12.30–−10.52)	**<** **.** **0001**	−13.54 (−15.29–−11.80)	**<**.**0001**	−10.13 (−11.13–−9.14)	**<**.**0001**	−4.02 (−4.61–−3.44)	**<**.**0001**
Female gender^b^	4.56 (1.86–7.26)	**0**.**0009**	6.98 (1.68–12.27)	**0**.**0099**	−0.69 (−3.61–2.23)	0.6412	1.38 (−0.46–3.22)	0.1416
Age	0.28 (0.18–0.37)	**<**.**0001**	0.35 (0.17–0.54)	**0**.**0002**	0.03 (−0.07–0.13)	0.5528	−0.12 (−0.18 –−0.05)	**0**.**0003**
Education^c^								
ISCED 3	1.12 (4.05–6.28)	0.6717	3.69 (−6.25–13.62)	0.4665	4.20 (−1.19–9.59)	0.1263	−0.28 (−3.68–3.12)	0.8719
ISCED 4,5	−1.82 (−7.45–3.80)	0.5249	−1.72 (−12.60–9.15)	0.7556	3.44 (−2.46–9.35)	0.2527	−1.54 (−5.27–2.19)	0.4187
Follow-up^d^								
6 months	−1.69 (−3.42–0.04)	0.0562	−5.02 (−8.50–−1.55)	**0**.**0047**	−2.95 (−5.02–−0.89)	**0**.**0051**	−1.33 (−2.47 –−0.20)	**0**.**0216**
12 months	−2.24 (−4.02 –−0.47)	**0**.**0132**	−7.93 (−11.48–−4.37)	**<**.**0001**	−4.05 (−6.16–−1.94)	**0**.**0002**	−2.79 (−3.96 –−1.63)	**<**.**0001**
24 months	−1.77 (−3.74–0.20)	0.0782	−7.26 (−11.21–−3.31)	**0**.**0003**	−3.92 (−6.27–−1.57)	**0**.**0011**	−3.02 (−4.32 –−1.71)	**<**.**0001**
History of cancer	1.88 (−1.94–5.70)	0.3338	0.94 (−6.72–8.59)	0.8106	5.28 (0.84–9.71)	**0**.**0198**	−2.44 (−4.96–0.08)	0.0572
History of asthma	5.09 (0.095–10.09)	**0**.**0458**	−0.89 (−10.61–8.82)	0.8567	1.56 (−3.81–6.93)	0.5687	1.96 (−1.41–5.32)	0.2538
History of COPD	2.46 (−3.09–8.02)	0.3842	11.05 (0.24–21.87)	**0**.**0452**	5.10 (−1.38–11.57)	0.1225	3.19 (−0.40–6.77)	0.0814
History of depression	0.23 (−3.73–4.20)	0.9084	−3.15 (−11.06–4.75)	0.4337	0.80 (−3.84–5.44)	0.7354	2.53 (−0.09–5.15)	0.0584
Smoking^e^								
Current smoker	0.91 (−3.92–5.74)	0.7124	0.83 (−8.66–10.32)	0.8639	−0.25 (−5.57–5.08)	0.9276	−0.82 (−4.05–2.42)	0.6216
Ex-smoker	0.16 (−2.36–2.69)	0.9004	−2.88 (−7.86–2.11)	0.2580	0.31 (−2.45–3.08)	0.8239	0.85 (−0.86–2.55)	0.3318
Body Mass Index [kg/m^2^]	0.24 (0.06–0.42)	**0**.**0101**	0.051(−0.31–0.41)	0.7820	0.13 (−0.07–0.33)	0.1960	0.13 (0.003–0.25)	**0**.**0439**
Symptoms of depression^f^	1.21 (0.83–1.58)	**<**.**0001**	1.13 (0.39–1.86)	**0**.**0027**	0.67 (0.25–1.09)	**0**.**0019**	0.56 (0.32–0.81)	**<**.**0001**
Symptoms of anxiety^f^	0.12 (−0.29–0.49)	0.5326	1.48 (0.75–2.22)	**<**.**0001**	0.44 (0.01–0.86)	**0**.**0431**	2.21 (1.97–2.46)	**<**.**0001**
Duration hospitalization	0.28 (0.14–0.41)	<.0001	0.38 (0.12–0.65)	**0**.**0046**	0.14 (−0.01–0.28)	0.0623	0.08 (−0.01–0.17)	0.0880
sPESI score ≥1^g^	1.76 (−0.86–4.38)	0.1879	1.36 (−3.77–6.49)	0.6024	0.31 (−2.51–3.13)	0.8288	0.53 (−1.25–2.31)	0.5592

Bold type indicates significant *p*-values.

CI, confidence interval; ISCED, international standard classification of education; COPD, chronic obstructive pulmonary disease; PE, pulmonary embolism; sPESI, simplified pulmonary embolism severity index.

^a^ Chronic Respiratory Questionnaire, continuous score.

^b^ Reference: men.

^c^ Reference: ISCED 1,2.

^d^ Reference: 3-months follow-up.

^e^ Reference: never smoker.

^f^ Hospital Anxiety and Depression Scale continuous subscale scores.

^g^ Reference: sPESI score = 0.

Additional analyses using the single PEmb-QOL items on dyspnea frequency and intensity confirmed the significant association of dyspnea with body mass index and symptoms of depression (see Supplementary Tables S2, S3), whereas FEV1 was not found to be significantly related with dyspnea frequency and intensity. History of asthma was significantly related with dyspnea frequency but not with intensity, and symptoms of anxiety were significantly related with dyspnea intensity, but not with dyspnea frequency.

### 3.4 Association between dyspnea and PE-specific HRQOL

The CRQ dyspnea score was independently and significantly associated with all PEmb-QOL subscales. High levels of dyspnea were associated with more “Activities of daily living limitations” (estimate −11.41, 95% CI −12.30 to −10.52, *p* < 0.0001), more “Work-related problems” (estimate −13.54, 95% CI −15.29 to −11.80, *p* < 0.0001), more “Social limitations” (estimate −10.13, 95% CI −11.13 to −9.14, *p* < 0.0001), and more “Emotional complaints” (estimate −4.02, 95% CI −4.61 to −3.44, *p* < 0.0001) (see Table 3). All models were adjusted for age, gender, education level, history of cancer, history of asthma, history of COPD, history of depression, smoking, body mass index, symptoms of anxiety and depression, duration of hospitalization, sPESI score, and follow-up time point.

When using the single PEmb-QOL items on dyspnea frequency and intensity as independent variables instead of the CRQ dyspnea score, the associations between dyspnea and all PEmb-QOL subscales remained significant (*p* < 0.0001).

## 4 Discussion

The present study showed that dyspnea—specifically dyspnea when walking or doing household chores—is a common problem in the time interval between 3 months and 2 years after acute PE. Even 2 years after acute PE only less than one half of the patients reported to be free of dyspnea. Furthermore, no significant change of dyspnea during the observation interval was found. High body mass index, symptoms of depression or anxiety, and FEV1 values were significantly associated with the CRQ dyspnea score. Furthermore, dyspnea was significantly and strongly associated with impairments of PE-specific HRQOL.

Depending on the measure used to assess dyspnea and the follow-up time, the present study showed that between 45% and 64% of the participants had dyspnea in some way with 13%–32% having at least moderate intensity of dyspnea. These results confirm findings from previous studies that dyspnea is a very common symptom in the post-acute phase of PE affecting up to 50% of the patients (11, 14, 15, 20, 21).

Besides the high prevalence of dyspnea, we also found no significant improvement of dyspnea over time. Consistent with these results, Farmakis et al. (15) found no significant improvement of the modified Medical Research Council dyspnea scale from 3–12 months after the acute PE event in 255 patients. Kahn at al (25). also reported that the strongest improvement of dyspnea happened in the first month after the acute PE. Farmakis et al. (15) supposed that the missing improvement of dyspnea despite an improvement of the 6-minute walking distance test among their study participants might have psychological reasons. This hypothesis may be supported by Fischer et al. (18) who found a significant association between dyspnea and symptoms of depression and anxiety in their analyses on the predictors of mental health problems after acute PE. However, so far no prior studies have investigated the contribution of symptoms of depression and anxiety to the persistence of dyspnea. Therefore, the results of the present study, where symptoms of depression and anxiety showed the strongest associations with dyspnea irrespective of other demographic and clinical variables are of utmost importance. The association between dyspnea and emotional or psychological states is not a new finding as a number of studies indicated that psychological states can cause or be caused by dyspnea (38, 39). Consequently, it seems reasonable to consider the multidimensional nature of dyspnea, involving the assessment of the sensory-perceptual domain, the affective distress and the symptom burden, in the post-PE patient management in the future (40). In addition, mental health problems such as anxiety and depression should be identified and treated early in the disease course (18).

In general, prior studies failed to identify a consistent set of clinical variables that predict dyspnea in patients with PE (20, 21, 25). Similarly, in the present study variables that indicate PE severity, such as sPESI score, duration of hospitalization, bilateral PE presentation, presence of central thrombi or infiltrates, showed no significant associations with dyspnea. Only low FEV1 values were significantly related with the CRQ dyspnea score, but not with the single items on dyspnea frequency and intensity, which may be a matter of small sample size or overadjustment in the statistical models.

High body mass index is the only variable that was consistently associated with dyspnea in several studies, including the present one (20, 21, 24, 25). Whether weight reduction could improve recovery and dyspnea after PE may be an interesting question for future research.

The present study showed that dyspnea was the strongest contributor to impaired HRQOL over the observation period of two years. Neither clinical factors nor socio-demographic characteristics or current mental health showed such a strong association with the subscales of the PEmb-QoL questionnaire. These results are in line with previous studies (11, 14, 23) and highlight the need for a comprehensive diagnosis and treatment of dyspnea in order to improve recovery and HRQOL, and to minimize the burden associated with acute PE (41).

### 4.1 Limitations

The main strengths of the present cohort study are the large sample of patients with a first episode of acute PE assessed four times between 3 months and 2 years after their acute PE with good response rates. Other available studies are mostly cross-sectional studies or follow-up studies with smaller sample sizes. Furthermore, the statistical models were adjusted for relevant confounders which were not considered in prior studies, for instance history of depression and current symptoms of depression and anxiety. Moreover, it is the first study which applied three measures of dyspnea in order to examine the validity of the statistical models.

However, some limitations should be considered. Since the instruments for the assessment of dyspnea and disease-specific HRQOL request information on the impairment in everyday life activities after the PE event, it was not appropriate to use them for an in-hospital baseline assessment. Consequently, a baseline assessment shortly after the acute PE is missing. Information about the presence of dyspnea prior to the acute PE event is also missing. However, such an effect might have been sufficiently considered by adjustment for history of asthma and COPD. Although many relevant confounders were included in the multivariable regression models, some potentially relevant confounders, e.g., pulmonary artery systolic pressure and diagnosis of chronic embolic pulmonary hypertension, were not assessed.

Despite excellent response rates above 90% in the first year of follow-up, the analyzed sample is not representative for the 977 patients initially included in the study. Many of those patients have disagreed in a follow-up and these are expected to be more severely ill than those patients who agreed to complete follow-up surveys. This selection bias may have also resulted in an underestimation of HRQOL impairments and dyspnea frequency and severity.

Patients were recruited from a single university hospital in southern Germany which may restrict external validity. Finally, due to the study design, no causal effects can be derived from the results.

### 4.2 Conclusions

In conclusion, the present study showed that dyspnea is a very common and persisting symptom that considerably impairs the patients' HRQOL and is strongly associated with symptoms of depression and anxiety. These results highlight the need for a comprehensive diagnostic and interventional strategy to improve dyspnea and the psychological factors, that may cause or maintain dyspnea.

## Data Availability

The data that support the findings of this study are available from the Chair of Epidemiology, Medical Faculty, University of Augsburg but restrictions apply to the availability of these data, which are not publicly available. Data are however available from the authors upon reasonable request and with permission of the Chair of Epidemiology. Requests to access the datasets should be directed to Christa.Meisinger@med.uni-augsburg.de.
