# Supplementary material for: Dyspnea after a first episode of pulmonary embolism: prevalence, predictors and long-term associations with health-related quality of life
Source: Front Cardiovasc Med. 2025 Jul 7;12:1595705. doi: 10.3389/fcvm.2025.1595705 (PMC12277268; doi:10.3389/fcvm.2025.1595705)
Supplement: Supplementary file 1 [file Table1.docx]

Supplementary Table 1: Variables associated with dyspnea according to the PEmb-QOL item on dyspnea frequency: results of the mixed models (n = 156, 449 observations)

|  | Dyspnea | | | |
| --- | --- | --- | --- | --- |
|  | Estimate | Lower CI | Upper CI | p-value |
| Female gender^2^ | -0.13 | -0.58 | 0.31 | 0.5554 |
| Age | 0.01 | 0.004 | 0.03 | 0.1211 |
| Education^3^ |  |  |  |  |
| ISCED 3 | 0.18 | -0.52 | 0.88 | 0.6165 |
| ISCED 4,5 | 0.36 | -0.41 | 1.13 | 0.3610 |
| Follow-up^4^ |  |  |  |  |
| 6 months | 0.007 | -0.19 | 0.20 | 0.9409 |
| 12 months | -0.02 | -0.22 | 0.18 | 0.8647 |
| 24 months | 0.10 | -0.12 | 0.32 | 0.3768 |
| History of cancer | 0.11 | -0.40 | 0.63 | 0.6619 |
| History of asthma | -1.18 | -1.96 | -0.40 | **0.0030** |
| History of COPD | -0.59 | -1.18 | 0.007 | 0.0527 |
| Smoking^5^ |  |  |  |  |
| Current smoker | -0.17 | -0.79 | 0.44 | 0.5788 |
| Ex-smoker | -0.08 | -0.42 | 0.27 | 0.6539 |
| Body Mass Index [kg/m^2^] | -0.04 | -0.06 | -0.02 | **0.0010** |
| Symptoms of depression^6^ | -0.08 | -0.13 | -0.03 | **0.0007** |
| Symptoms of anxiety^6^ | -0.04 | -0.09 | 0.001 | 0.0574 |
| Duration hospitalization | -0.01 | -0.04 | 0.01 | 0.2774 |
| sPESI score ≥1^7^ | 0.06 | -0.30 | 0.43 | 0.7335 |
| Bilateral PE localization | 0.19 | -0.39 | 0.76 | 0.5262 |
| Central thrombi | 0.32 | -0.07 | 0.70 | 0.1046 |
| Infiltrates | 0.02 | -0.36 | 0.39 | 0.9363 |
| FEV_1_ [l] | 0.18 | -0.11 | 0.46 | 0.2210 |

CI: confidence interval; ISCED: International Standard Classification of Education; COPD: chronic obstructive pulmonary disease; PE: pulmonary embolism; sPESI: Simplified Pulmonary Embolism Severity Index;

^1^Chronic Respiratory Questionnaire, continuous score; ^2^Reference: men; ^3^Reference: ISCED 1,2; ^4^Reference: 3-months follow-up; ^5^Reference: never smoker; ^6^Hospital Anxiety and Depression Scale continuous subscale scores; ^7^Reference: sPESI score = 0
