# Supplementary material for: Dyspnea after a first episode of pulmonary embolism: prevalence, predictors and long-term associations with health-related quality of life
Source: Front Cardiovasc Med. 2025 Jul 7;12:1595705. doi: 10.3389/fcvm.2025.1595705 (PMC12277268; doi:10.3389/fcvm.2025.1595705)
Supplement: Supplementary file 2 [file Table2.docx]

Supplementary Table 2: Variables associated with dyspnea according to the PEmb-QOL item on dyspnea intensity: results of the mixed models (n = 159, 466 observations)

|  | Dyspnea | | | |
| --- | --- | --- | --- | --- |
|  | Estimate | Lower CI | Upper CI | p-value |
| Female gender^2^ | 0.18 | -0.24 | 0.61 | 0.3986 |
| Age | -0.004 | -0.02 | 0.01 | 0.6591 |
| Education^3^ |  |  |  |  |
| ISCED 3 | -0.23 | -0.90 | 0.44 | 0.4962 |
| ISCED 4,5 | -0.44 | -1.17 | 0.29 | 0.2365 |
| Follow-up^4^ |  |  |  |  |
| 6 months | 0.15 | -0.03 | 0.33 | 0.0957 |
| 12 months | 0.006 | -0.19 | 0.18 | 0.9532 |
| 24 months | 0.03 | -0.23 | 0.17 | 0.7460 |
| History of cancer | 0.03 | -0.44 | 0.50 | 0.8947 |
| History of asthma | 0.72 | -0.02 | 1.45 | 0.0566 |
| History of COPD | 0.42 | -0.13 | 0.97 | 0.1310 |
| Smoking^5^ |  |  |  |  |
| Current smoker | 0.18 | -0.40 | 0.75 | 0.5487 |
| Ex-smoker | 0.30 | -0.11 | 0.53 | 0.1908 |
| Body Mass Index [kg/m^2^] | 0.03 | 0.007 | 0.05 | **0.0092** |
| Symptoms of depression^6^ | 0.08 | 0.04 | 0.13 | **0.0002** |
| Symptoms of anxiety^6^ | 0.05 | 0.009 | 0.09 | **0.0165** |
| Duration hospitalization | 0.01 | -0.009 | 0.03 | 0.2437 |
| sPESI score ≥1^7^ | -0.12 | -0.47 | 0.23 | 0.5090 |
| Bilateral PE localization | -0.52 | -1.07 | 0.02 | 0.0608 |
| Central thrombi | -0.14 | -0.51 | 0.22 | 0.4338 |
| Infiltrates | -0.03 | -0.37 | 0.32 | 0.8795 |
| FEV_1_ [l] | -0.12 | -0.39 | 0.15 | 0.3867 |

CI: confidence interval; ISCED: International Standard Classification of Education; COPD: chronic obstructive pulmonary disease; PE: pulmonary embolism; sPESI: Simplified Pulmonary Embolism Severity Index;

^1^Chronic Respiratory Questionnaire, continuous score; ^2^Reference: men; ^3^Reference: ISCED 1,2; ^4^Reference: 3-months follow-up; ^5^Reference: never smoker; ^6^Hospital Anxiety and Depression Scale continuous subscale scores; ^7^Reference: sPESI score = 0
