# Supplementary figures and images for: Dyspnea after a first episode of pulmonary embolism: prevalence, predictors and long-term associations with health-related quality of life
Source: Front Cardiovasc Med. 2025 Jul 7;12:1595705. doi: 10.3389/fcvm.2025.1595705 (PMC12277268; doi:10.3389/fcvm.2025.1595705)

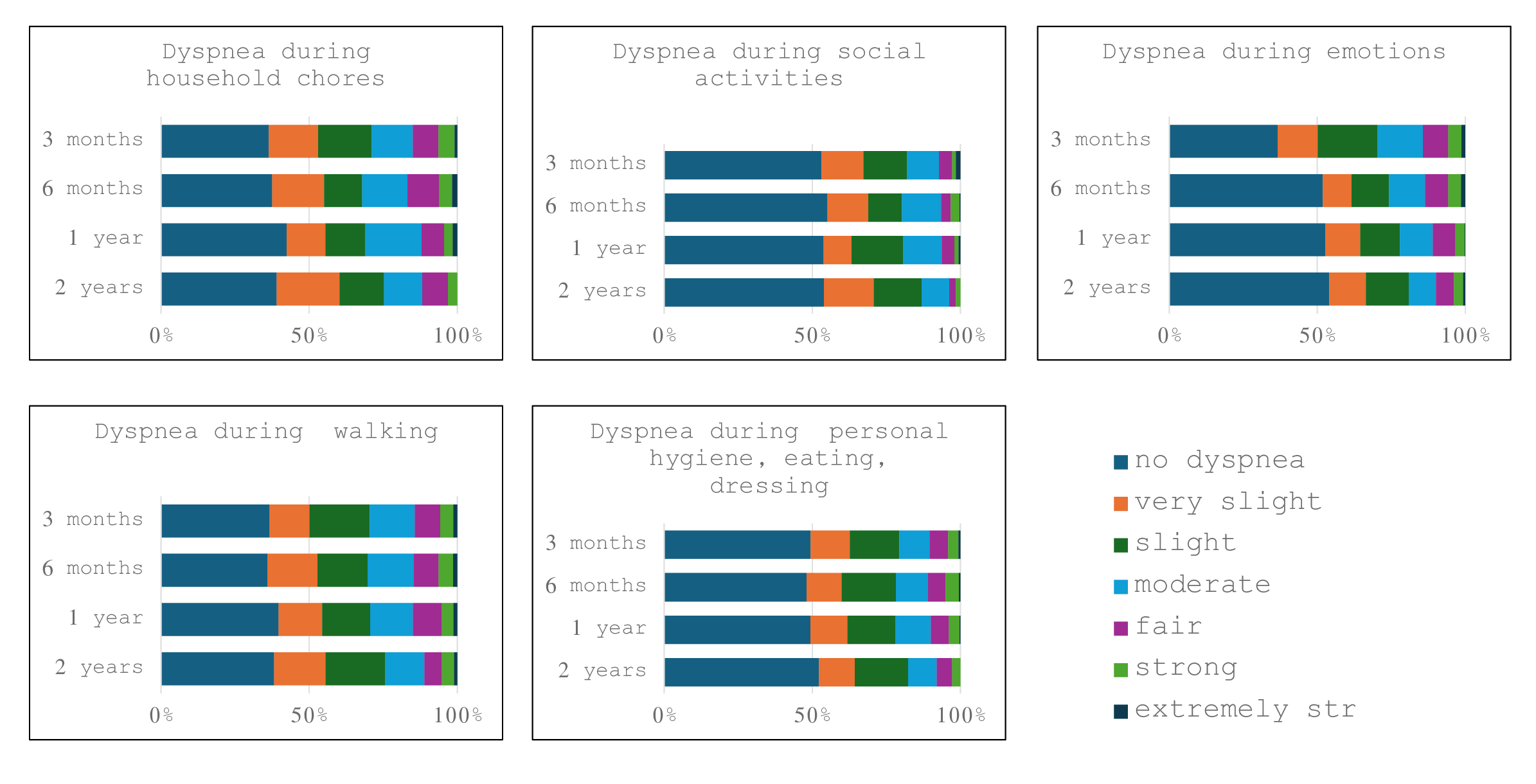

Supplement: Supplementary file 3 [file Image1.tiff]

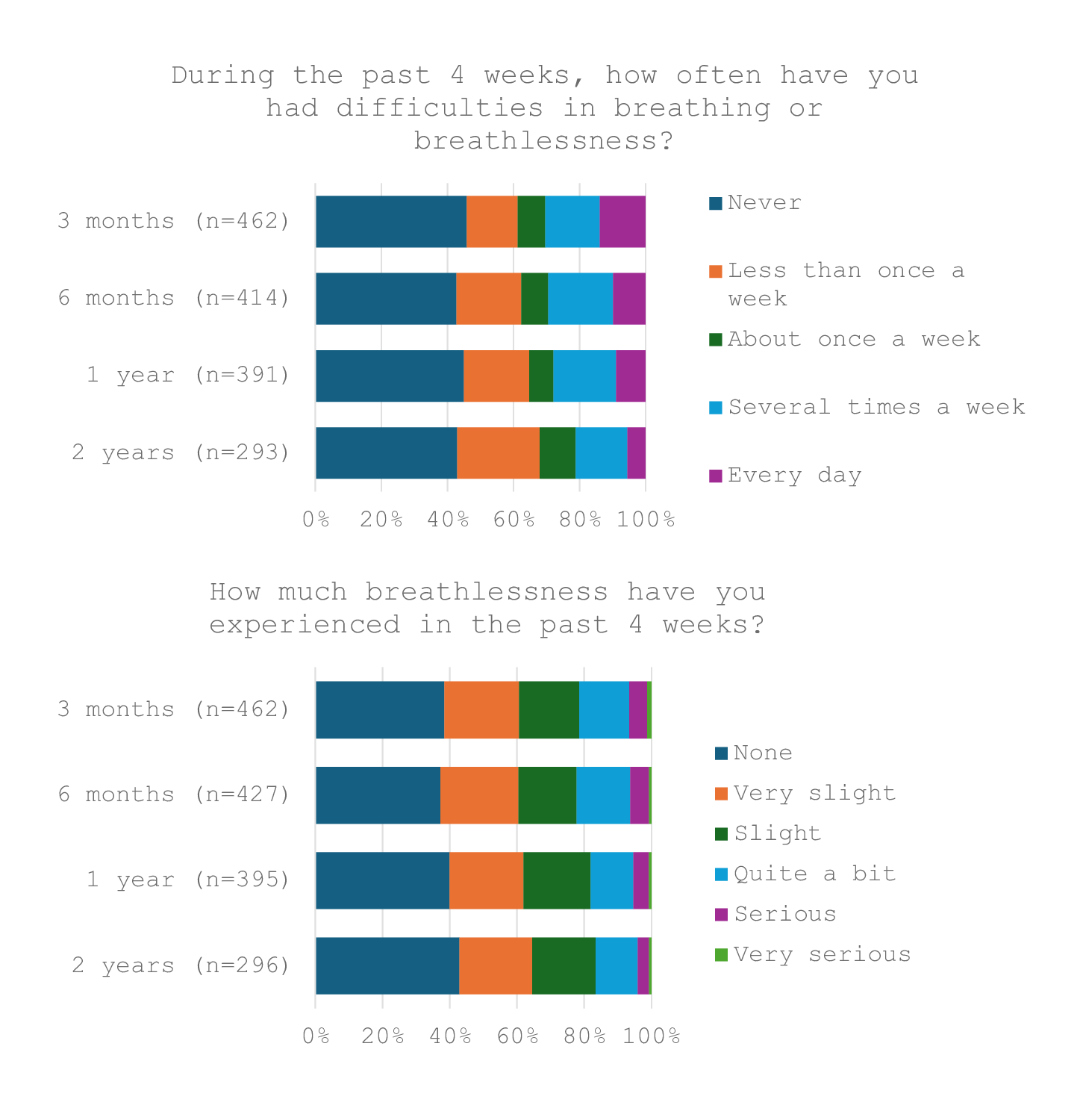

Supplement: Supplementary file 4 [file Image2.tiff]
